# Feasibility of early radial artery occlusion recanalization and reuse through transradial access for neuroendovascular procedures

**DOI:** 10.1186/s12883-024-03549-8

**Published:** 2024-01-31

**Authors:** Ranze Cai, Yingchuang Jiang, Jian Wu, Qiuping Li, Biao Qi

**Affiliations:** 1 Department of Neurosurgery, Zhongshan Hospital, Fudan University (Xiamen Branch), Xiamen, Fujian Province 361006 China; 2 Department of Neurosurgery, Zhongshan Hospital, Fudan University, Shanghai, 200032 China

**Keywords:** Transradial access TRA, Radial artery occlusion RAO, Neuroendovascular procedures

## Abstract

**Background:**

Radial artery occlusion (RAO) remains a significant limitation of neuroendovascular procedures peformed through transradial access (TRA) when radial artery needs to be reused. Instances of early RAO recanalization to successfully complete neuroendovascular procedures have been rarely documented.

**Materials and methods:**

Documents and imaging data were extracted retrospectively for all patients who underwent TRA diagnostic angiography and neuroendovascular procedures in our center from June 2022 to February 2023. The patients with early RAO who required repeat TRA were included.

**Results:**

A total of 46 patients underwent repeat TRA, and 13 consecutive patients who experienced early RAO after angiography as confirmed by ultrasonography were enrolled in this study. The occluded radial arteries were successfully recanalized, and subsequent neuroendovascular procedures were carried out successful. During an average follow-up time of 7.1 months, no patients exhibited symptomatic RAO, dissection, hematoma or pseudoaneurysm.

**Conclusions:**

Early RAO recanalization and reused for neuroendovascular procedures through TRA is feasible. A visually guided and stable puncture process plays a crucial role in successfully recanalizing early RAO.

## Background

The adoption of transradial access (TRA) for diagnostic cerebral angiography and neuroendovascular procedures is increasing worldwide. This trend may be attributable in part to the recognition that TRA offers substantial reductions in complications and costs when compared to transfemoral access (TFA) [1–4]. Numerous studies have demonstrated the feasibility of repeat TRA for neuroendovascular procedures [5, 6]. However, despite the many advantages of TRA, RAO remains a significant limitation of neuroendovascular procedures via TRA when radial artery needs to be reused. Nevertheless, reaccessing the occluded radial artery is a feasible approach for performing repeated neuroendovascular procedures [7]. In this retrospective study, we present our experience and techniques in recanalizing early RAO through repeat TRA for neuroendovascular procedures at a single center.

## Methods

### Patients

The electronic medical records (inpatient and outpatient) of all patients who underwent diagnostic angiography and interventional neuroendovascular procedures utilizing TRA at our center from June 2022 to February 2023 were retrospectively reviewed. Inclusion criteria were: (1) Patients underwent diagnostic angiography or interventional neuroendovascular procedures via TRA; (2) Patients were diagnosed with early RAO through vascular ultrasound examination; (3) Patients underwent repeated TRA on the same side. Exclusion criteria were: (1) Patients initially underwent TRA followed by unscheduled TFA; (2) Patients underwent diagnosis and treatment of non-cerebrovascular diseases via TRA. The study was approved by the Ethics Committee of Zhongshan Hospital, Fudan University (Xiamen Branch). The need for informed consent was waived by ethics committee of Zhongshan Hospital (Xiamen branch) due to retrospective nature of the study. All investigations were performed in accordance with the Declaration of Helsinki.

### Recanalization technique

**Fig. 1:**
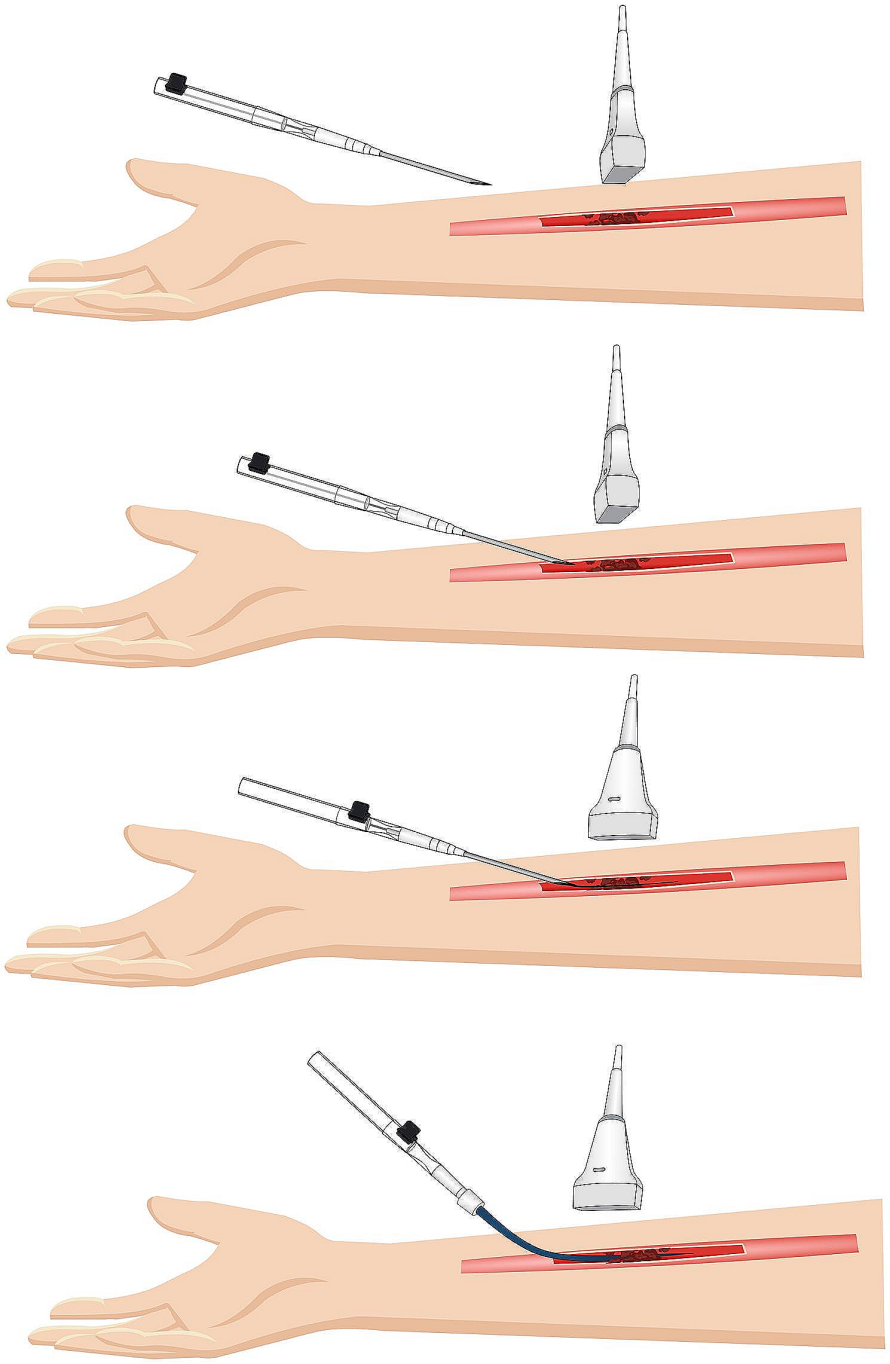
The process of puncture with arrow quick flash radial artery catheterization set

We confirmed thrombosis and the absence of blood flow through the radial artery thrombosis by ultrasound within 48 h after the patient’s angiography was completed. The repeated puncture site was located within a 3 cm radius around the initial puncture site. Ultrasound examination revealed the presence of thrombus in the radial artery, and compression of the ultrasound probe did not demonstrate any vascular deformation or blood flow. We utilized an Arrow Quick Flash radial artery catheterization set (RA-04220, Mexico) for the puncture needle. The set consisted of a puncture needle tube encompassed by an outer sheath and an internal movable guide wire (Fig. 1).

**Fig. 2:**
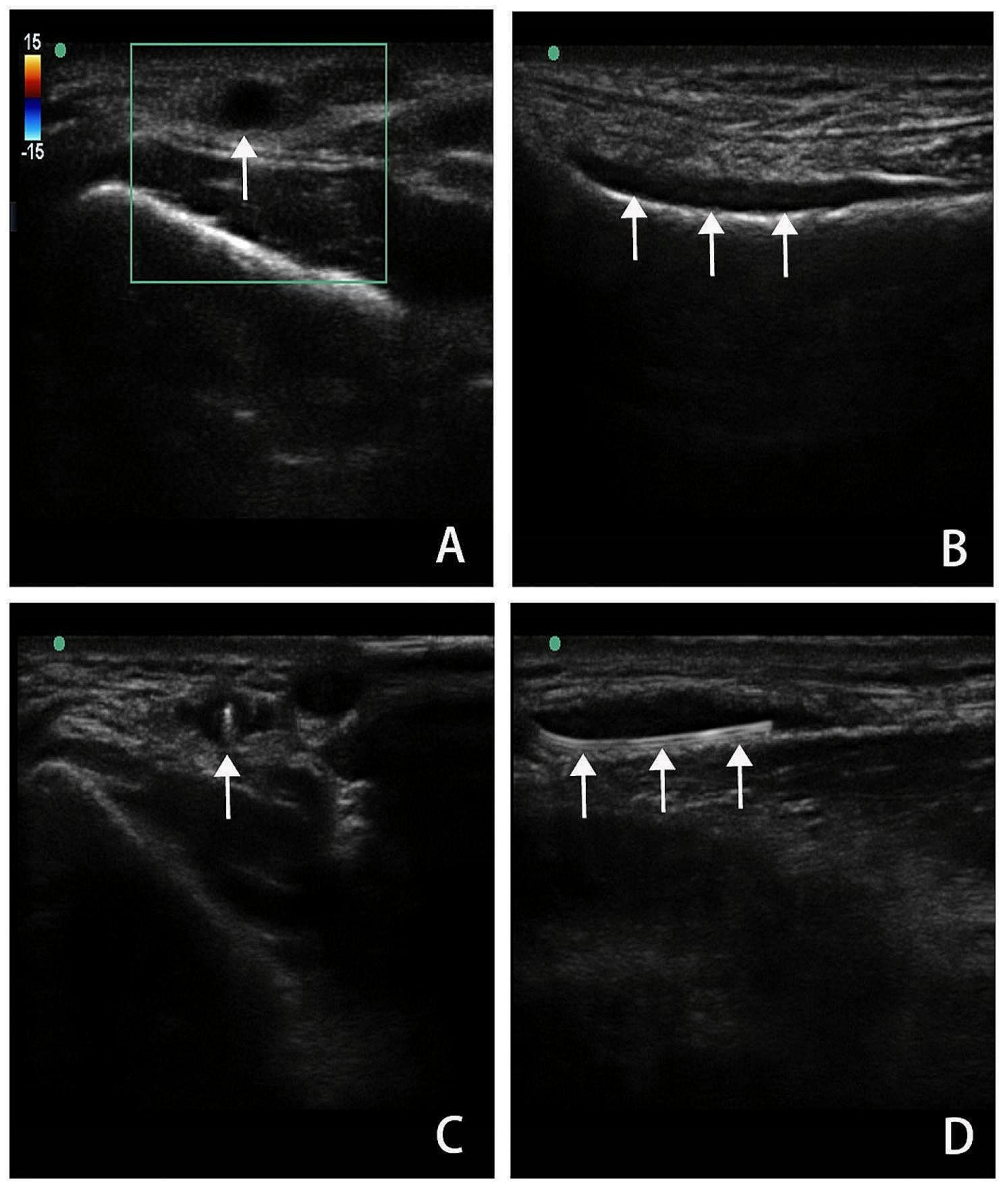
Ultrasound images showing transverse (**A**, ↑ black circle was radial artery) and long-axis (**B**, ↑ black fusiform was radial artery) of the radial artery with RAO. The radial artery with the echogenic needle tip within the lumen in transverse (**C**, ↑ white dot in the round black was needle tip). The movable guide wire was located in the true lumen through ultrasound (**D**, ↑ white strip was movable guide wire)

Once the echogenic tip of the puncture needle was visualized within the lumen of the radial artery under transverse ultrasound guidance, the movable guide wire was slowly advanced into the blood vessel and positioned within the true lumen of the blood vessel through long-axis ultrasound (Fig. 2).

The outer sheath was then carefully advanced, observing bleedback and noting only a small amount of blood was found around the outer sheath. To confirm its position within the radial artery, radial arteriography was performed, excluding cases with tortuous arteries, associated calcified plaques, adjacent small dissections, and long segment clots. Following confirmation, a cocktail of verapamil (2 mg), nitroglycerin (100 µg), and 5 mL of aspirated blood was injected. Under the guiadance of the roadmap technique, the guide wire of either 4 Fr or 6 Fr introducers was navigated into the radial artery and successfully placed. To prevent clot formation, heparin (5000 U) was added to physiological saline for instillation during cerebral angiography. The radial artery compression device (TR Band, Japan) has been used at the end of the diagnostic procedure.

### Statistical analysis

SPSS version 24.0 software (SPSS Inc, Chicago, IL) was used for the analysis. Continuous variables were presented as mean ± standard deviation. The time of early RAO recanalization are presented as median and interquartile range. The intergroup comparison was performed using t-test. Unless stated otherwise, a 2-tailed *P* < 0.05 was considered statistically significant.

## Results

A total of 144 patients underwent TRA procedures for diagnostic angiography (127 cases) and neuroendovascular procedures (62 cases) from June 2022 to February 2023, resulting in a total of 189 TRA instances. Among these, diagnostic angiography was performed from the left TRA in 2 cases, while the remaining 142 cases were conducted on the right side. All repeated TRA were performed on the right side. The initial planned TRA count was 193, but two diagnostic angiographies and two neuroendovascular procedures were excluded due to the intraoperative change from TRA to TFA. One of the excluded cases involved an elderly woman with right ophthalmic artery stenosis of the internal carotid artery, who was diagnosed with RAO after cerebral and coronary angiography at other hospitals using a 4 Fr sheath via TRA. Four days after the initial operation, the patient was transferred to our center, where RAO was recanalized, and a 6 Fr sheath was successfully placed. Angiography revealed that the patient’s dominant artery in the right hand was the ulnar artery, and the radial artery was unable to accommodate the 6 Fr sheath, ultimately requiring a switch to TFA.

Over the course of the 8-month study, a total of 46 patients underwent repeat TRA for diagnostic angiography, intervention, or postoperative recheck. In diagnostic angiography, all patients received a 4 Fr or 5 Fr sheath, while intervention procedures involved the placement of a 6 Fr sheath. Among the patients, 44 had a successful second TRA, and 2 underwent a third successful TRA. During the initial diagnostic puncture procedures, ultrasound guidance wasn’t commonly employed unless there were multiple unsuccessful attempts at puncturing. A total of 13 patients among the 46 requiring a second procedure were diagnosed with early RAO based on ultrasonography following their initial diagnostic angiography. The average diameter of the radial artery in 13 cases of RAO patients was 2.1 ± 0.4 mm, while in 33 cases without RAO, the average diameter was 2.3 ± 0.4 mm. The diameter of the radial artery was not correlated with the risk of occlusion (*P* > 0.05). All 13 patients underwent recanalization of the occluded radial artery within 6 days (IQR: 3–7 days), and subsequent intervention procedures were then performed successfully (Table 1).

**Table 1:** Characteristics of patients with RAO recanalization and reuse through TRA

Patient characteristics	Number of patients (N = 13)
Mean age, y	54 ± 17.3
≤ 54 y	5 (38.5%)
>54 y	8 (61.5%)
Sex	/
Female	7 (53.8%)
Male	6 (46.2%)
Mean diameter of radial artery, mm	2.1 ± 0.4
≤2.1 mm	6 (46.2%)
>2.1 mm	7 (53.8%)
Pathology Aneuysm Arteriovenous malformation Internal carotid occlusion Procedure performed Aneurysm repair (coil) Aneurysm repair (coil + stent) Liquid embolization Angioplasty carotid stent	/ 9 (69.2%) 2 (15.4%) 2 (15.4%) / 1 (7.7%) 8 (61.5%) 2 (15.4%) 2 (15.4%)
TRA times	/
2	11 (84.6%)
3	2 (15.4%)
6 months ultrasound examination	/
RAO	4(30.8%)
No RAO	9 (69.2%)

RAO: radial artery occlusion; TRA: transradial access

Nine patients (9/13, 69.2%) did not experience recurrent RAO following successful recanalization and reuse, while four (4/13, 30.8%) exhibited reappearance of RAO after the intervention procedures. No complications such as forearm pain, radial artery dissection, hand ischemia, or forearm numbness were observed during the 7.1-months follow-up period.

## Discussion

TRA has increasingly become a standard approach for diagnostic cerebral angiography and neuroendovascular procedures. When compared to TFA, TRA offers the advantage of reducing the risk of access-site complications [3]. At our center, the right TRA was predominantly preferred due to its convenience for the surgeons. Additionally, based on our single-center experience, the right TRA has demonstrated greater stability for cerebrovascular diseases, particularly for certain left carotid system diseases, except for cases involving the left vertebral artery.

The reported incidence of RAO varies from 0.8 to 33%, with early RAO occuring at approximately 5.5–7.7% [8–10]. Several factors have been associated with RAO, including multiple puncture failures, female sex, age, vessel diameter, introducer sheath size, prolonged post-procedure compression time, patient hemostasis, and higher doses of anticoagulation [11, 12]. In our center, we have implemented the use of a radial artery compression device (TR Band, Japan, max air: 18 mL). However, it is worth noting that prolonged compression time (6 h), deflated by 2 mL every 2 h and high-pressure compression (14 mL) might contribute to the incidence of RAO in our center. 2 mL deflation every 15 min (rather than every 2 h) is much more practical and feasible, even in cases involving large sheaths and patients on antiplatelets therapy. Considering the dosage of heparin and the patient’s antiplatelet status, the duration of wrist band retention after diagnostic angiography is typically 1–2 h, while it is extended to 3–4 h following treatment procedures [13].

Futhermore, studies have demonstrated the safety and feasibility of recanalizing chronic radial artery occlusion through distal TRA [14, 15]. In 2021, Feng Li et al. successfully recanalized a RAO formed three days post-operation using distal TRA guided by vascular ultrasonography [16]. Neil Majmundar et al. reported their successful experience in repeat TRA procedures and techniques for reaccessing occluded arteries in selected patients. Among their nine RAO patients, five failed to recanalize RAO and were subsequently transferred to TFA, while four cases achieved successful recanalization [7]. Utilizing our method, we achieved a 100% technological success rate in early RAO recanalization. Notably, in comparison to previous studies, the early RAO formation site in this study was primarily near the puncture point, resulting in relatively shorter distances of occlusion. After the needle reaches the center of the blood vessel, we maintained stability in the right hand while using the left hand to operate the ultrasound and advance the movable guide wire of the puncture needle and outer sheath. This technique is crucial to avoid exchanging the left and right hands. In contrast to a micro puncture needle, the Arrow quick flash device avoids the need to exchange the right and left hands to place the guidewire, thus ensuring stability.

Ultrasound currently plays a unique role in diagnosing RAO and guiding radial artery puncture, offering the benefit of a visible puncture process and the ability to assess the compatibility between the radial artery and the outer diameter of the arterial sheathbefore the procedure [17]. Under ultrasound guidance, we can observe the tissue structure surrounding the radial artery, assess the length of thrombosis within the radial artery, and ensure that blood vessel lumen remains patent when pressure is applied to the ultrasound probe. Additionally, successful puncture of the outer sheath typically results in minimal bleeding, as observed in most medical records. Through the use of ultrasound imaging and manual injection of contrast agent, we confirm the stability of the outer sheath within the radial artery and assess blood flow conditions at the distal end of the sheath.

The early RAO recanalization through in situ puncture holds paramount significance in contemporary medical interventions. This approach encompasses a multifaceted spectrum of benefits and applications that underscore its pivotal role in clinical practice: (1) Facilitating subsequent interventional neuroendovascular or cardiovascular procedures after recanalization, allowing for comprehensive patient treatment; (2) Mitigating potential vascular injury from new approaches via repeated access routes, and reducing potential risks; (3) Offering a viable alternative for patients declining TFA, catering to individual preferences and enhancing patient satisfaction; (4) Addressing symptomatic RAO; (5) Providing arteriovenous fistula access for hemodialysis or serving as donors for coronary artery bypass grafting; (6) Achieving radial artery recanalization without the necessity for additional balloons or stents, and cutting down on costs.

There are several limitations to our single-center study. This study is a single-center retrospective design, which lacks randomization and presents challenges in controlling variables, thus susceptible to biases and confounding factors. Due to the small size of the sample used in our study, there are constraints that hinder the ability to conduct additional and more in-depth statistical analyses. Additionally, our study may not encompass the various situations encountered, such as patients with tortuous artery, associated calcified plaques, adjacent small dissections, or long segment clot etc. Therefore, further evaluation of this technology on a larger scale is necessary to validate its advantages and ensure its safety. Compared to the success rate of early RAO recanalization, the success rate in later-stage RAO appears relatively diminished, likely attributable to the presence of mature thrombi and longer thrombotic segments. Investigating these underlying factors and improving the success rate of recanalization will be a focus of our forthcoming research. Recanalization of RAO is a significant area of focus. However, it is crucial to carefully weigh the risks versus the benefits associated with the procedure.

## Conclusions

The recanalization of early RAO and subsequent neuroendovascular procedures through TRA are feasible. A visual and stable puncture process significantly contributes to successful recanalization of early RAO. Some patients did not experience recurrent RAO following recanalization and reuse procedure and even in cases where RAO developed postoperatively, no access-related asymptomatic issues were observed.

## Data Availability

The datasets used and/or analysed during the current study are available from the corresponding author on reasonable request.

## Abbreviations

TRA: transradial access
RAO: radial artery occlusion
TFA: transfemoral access
IQR: interquartile range
